# Infrared Spectra and Phototransformations of *meta*-Fluorophenol Isolated in Argon and Nitrogen Matrices

**DOI:** 10.3390/molecules27238248

**Published:** 2022-11-26

**Authors:** A. J. Lopes Jesus, Juracy Regis de Lucena Júnior, Rui Fausto, Igor Reva

**Affiliations:** 1 CQC-IMS, Faculty of Pharmacy, University of Coimbra, 3004-295 Coimbra, Portugal; 2 CCT, Department of Chemistry, State University of Paraíba, Campina Grande 58429-500, Brazil; 3 CQC-IMS, Department of Chemistry, University of Coimbra, 3004-535 Coimbra, Portugal; 4 CIEPQPF, Department of Chemical Engineering, University of Coimbra, 3030-790 Coimbra, Portugal

**Keywords:** *meta*-fluorophenol, matrix isolation, infrared spectroscopy, quantum-chemistry calculations, hydrogen atom tunneling, vibrationally-induced chemistry, UV-induced photochemistry

## Abstract

Monomers of *meta*-fluorophenol (*m*FP) were trapped from the gas phase into cryogenic argon and nitrogen matrices. The estimated relative energies of the two conformers are very close, and in the gas phase they have nearly equal populations. Due to the similarity of their structures (they only differ in the orientation of the OH group), the two conformers have also similar predicted vibrational signatures, which makes the vibrational characterization of the individual rotamers challenging. In the present work, it has been established that in an argon matrix only the most stable *trans* conformer of *m*FP exists (the OH group pointing away from the fluorine atom). On the other hand, the IR spectrum of *m*FP in a nitrogen matrix testifies to the simultaneous presence in this matrix of both the *trans* conformer and of the higher-energy *cis* conformer (the OH group pointing toward the fluorine atom), which is stabilized by interaction with the matrix gas host. We found that the exposition of the cryogenic N_2_ matrix to the Globar source of the infrared spectrometer affects the conformational populations. By collecting experimental spectra, either in the full mid-infrared range or only in the range below 2200 cm^−1^, we were able to reliably distinguish two sets of experimental bands originating from individual conformers. A comparison of the two sets of experimental bands with computed infrared spectra of the conformers allowed, for the first time, the unequivocal vibrational identification of each of them. The joint implementation of computational vibrational spectroscopy and matrix-isolation infrared spectroscopy proved to be a very accurate method of structural analysis. Some mechanistic insights into conformational isomerism (the quantum tunneling of hydrogen atom and vibrationally-induced conformational transformations) have been addressed. Finally, we also subjected matrix-isolated *m*FP to irradiations with UV light, and the phototransformations observed in these experiments are also described.

## 1. Introduction

**Scheme 1:**
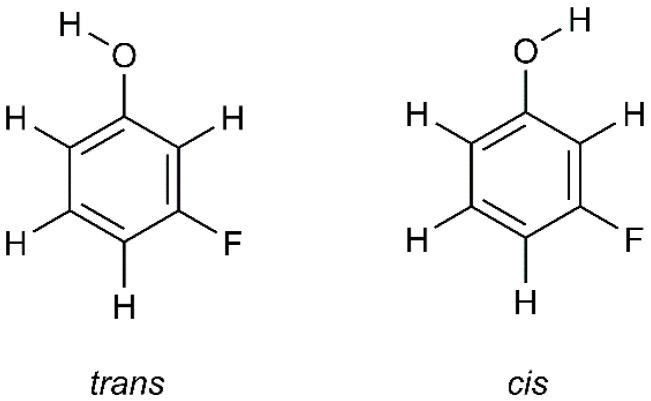
Molecular structures of the *trans* and *cis* conformers of *m*FP.

*meta*-Fluorophenol (*m*FP) is one of the three positional isomeric forms of fluorophenol (the others are *ortho*- and *para*-fluorophenol). This molecule can adopt two stable conformations, which can be designated as *cis* and *trans*, having the O–H group directed toward or away from the halogen substituent, respectively (see Scheme 1).

In the crystalline state, the *m*FP molecules adopt a non-planar *cis* conformation (an HOCC dihedral angle equal to 20.8°) and interact through OH⋯OH hydrogen bonds to form chains along the crystallographic 2_1_ screw axis [1]. This packing motif is stabilized by C–H⋯F close contacts. Regarding the isolated molecule, previous theoretical calculations carried out at DFT and MP2 levels of theory [2,3,4,5] revealed that the energy difference between the two rotamers does not exceed 0.84 kJ mol^−1^, with the *trans* form being more stable. Such a small energy difference permitted the detection of both conformers in the gas phase by microwave spectroscopy [5,6,7]. Moreover, based on their distinct electric dipole moments, they could be spatially separated by electrostatic deflection for the molecular gas seeded in helium or neon supersonic expansions [4]. ^1^H NMR experiments have also been performed for *m*FP dissolved in different aprotic solvents at 305 K [8]. In the obtained data, the *trans*/*cis* equilibrium constants were determined to fall between 2.3 and 2.9, depending on the solvent and on the concentration, corresponding to abundances of the most stable *trans* rotamer of 70–74%, which agrees with its higher polarity.

Previous IR spectroscopic studies have been undertaken for *m*FP in the neat liquid [9] and vapor phases [10,11], as well as in cyclohexane and acetonitrile dilute solutions [2,11]. Analysis of the 350−150 cm^−1^ region of the far-IR spectrum of the gaseous compound led to the identification of two bands due to OH torsion, at 319 and 311 cm^−1^, which were tentatively assigned to the *cis* and *trans* rotamers, respectively [10,11]. With regard to the spectra recorded in solutions, due to the broadening and overlapping of bands, only a very small number of absorptions could be assigned to a specific conformer by comparison with the computed vibrational data. In concordance with the ^1^H NMR data, the *trans* form was found to be the dominant conformer in solution, with an estimated abundance of 67%. IR spectra of *m*FP complexed with water [12], benzene [13], and carbon monoxide [14] molecules have also been measured under matrix isolation conditions. In spite of the existing previous few reports dealing with IR spectroscopic data of *m*FP, to the best of our knowledge, the detailed assignment of the spectrum of the monomeric compound has never been undertaken hitherto.

In the present investigation, mid-IR spectra of *m*FP were recorded after isolating monomers of this compound in low-temperature Ar and N_2_ matrices. Taking advantage of the high resolution of the matrix-isolation IR spectra, separation of the spectral signatures of the *cis* and *trans* conformers was successfully achieved with the aid of results of broadband IR-excitations carried out for the matrix-isolated compound and of computational calculations performed for both conformers by using DFT(B3LYP), MP2, and QCISD quantum chemical methods. Furthermore, the H-tunneling decay converting the higher-energy into the lower-energy OH rotamer of *m*FP, as well as the phototransformations induced by narrowband UV light with *λ* = 280–270 nm were also investigated.

## 2. Results and Discussion

### 2.1. Conformers of mFP and Energy Barriers for Their Interconversion

Table 1 summarizes the results of the quantum chemical calculations carried out for the *cis* and *trans* conformers of the *m*FP molecule at the B3LYP/aug-cc-pVTZ, MP2/aug-cc-pVTZ and QCISD/aug-cc-pVDZ levels (Cartesian coordinates of the fully optimized geometries are given in Table S1). To maximize the conjugation between the electron lone pairs of the oxygen atom and the π-system of the aromatic ring, both conformers have the hydroxyl group coplanar with the phenyl ring, thus belonging to the *C*_s_ symmetry point group. Since in the *trans* conformer the O–H and C–F bond dipoles are parallel, its computed electric dipole moment (*µ* = 2.7–3.1 D) is significantly larger than that predicted for the *cis* form (*µ* = 0.7–0.8 D), in agreement with previously reported data [4,5].

According to the calculations, the differences of electronic (Δ*E*_el_), zero-point-corrected electronic (Δ*E*_0_), and Gibbs (Δ*G*) energies between the two conformers amount to 0.58–0.70, 0.69–0.75, and 0.70–0.73 kJ mol^−1^, respectively, in favor of the *trans* form. The similar values of Δ*E*_el_, Δ*E*_0_, and Δ*G* reveal that the zero-point vibrational energy, as well as the thermal and entropic corrections, have a minor influence on the relative stability of the conformers. Our results are in agreement with those derived from computations performed at other levels of theory, with relative energies falling between 0.50 and 0.84 kJ mol^−1^ [2,3,4,5]. Energy differences not substantially different have also been estimated from the OH torsional potential based on far-infrared spectroscopic data (1.01 kJ mol^−1^) [10] and from the relative intensities of the rotational transitions observed for the two conformers in the microwave spectrum of the gaseous compound (1.10 kJ mol^−1^) [6]. The small energy gap between the two conformers is not surprising, owing to the large spatial separation between the fluorine and hydroxyl substituents, which precludes interaction between them. Among the fluorophenol positional isomers, such an interaction only takes place in the 2-fluorophenol analog, where the two substituents occupy vicinal positions [3,15].

The results of a Natural Bond Orbital (NBO) analysis carried out for the *m*FP molecule by Zeoly et al. [3] reveal that the greater stabilization of the *trans* conformer over the *cis* form is attributed to more favorable hyperconjugative stabilization in the former than in the latter conformer. Assuming a Boltzmann distribution based on the values of Δ*G* estimated at 298.15 K, the *trans*:*cis* population ratio of *m*FP was estimated to be around 57%:43%. This population ratio could be expected to be present in the *m*FP/Ar and *m*FP/N_2_ gaseous mixtures prior to the deposition of the matrices.

The energy barriers interconnecting the two *m*FP conformers (Δ*E*_el_^‡^) were calculated at the B3LYP and MP2 levels and have been included in Table 1 (the B3LYP potential energy profile for the OH rotamerization is shown in Figure S1). The values computed for the *cis* → *trans* and *trans* → *cis* isomerizations amount to 14.3–15.9 and 15.0–16.5 kJ mol^−1^, respectively, which are close to those estimated from torsional data in the gas phase [10] and cyclohexane solution [11] (15.4–16.6 kJ mol^−1^). It is well known that in a matrix environment at temperatures as low as those used in our experiments (14–16 K), activation energies of this order of magnitude are too large so that the isomerization could occur by means of an over-the-barrier mechanism [16,17,18]. Nevertheless, for various phenol derivatives with the two OH rotamers separated by energy barriers (in the direction of conformational relaxation) ranging from 10 to 20 kJ mol^−1^, it was not possible to identify spectral signatures of the higher-energy rotamers in freshly deposited matrices of noble gases, in spite of their non-negligible gas phase population [19,20,21,22,23,24,25,26]. Non-detection of these less stable rotamers has been attributed to their fast decay to the lower-energy counterparts by hydrogen atom quantum tunneling through the OH torsional barrier, a phenomenon that has been also identified for several carboxylic acids [27,28,29,30,31,32] and amino acids [33,34,35]. Spectroscopic characterization of the short-lived conformers has however been made possible by using nitrogen as the host matrix, which is found to reduce substantially the rate of the tunneling-induced OH flip as compared to noble gas matrices [22,23,27,28,34,35,36,37,38,39]. Based on these previously reported experimental findings, it is most likely that in a low-temperature Ar matrix, only the *trans* conformer of *m*FP should be spectroscopically detected. Hence, in the present work, as described in Section 2.2, in addition to the study of *m*FP isolated in an argon matrix, the compound was also isolated in solid N_2_ in an attempt to experimentally observe the higher-energy *cis* conformer.

### 2.2. Infrared Spectra of Matrix-Isolated mFP

**Figure 1:**
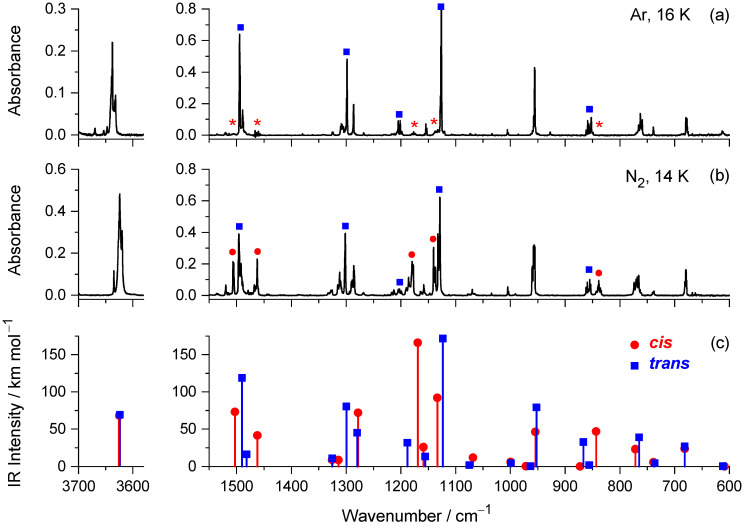
Experimental mid-IR spectra of *m*FP recorded after isolating monomers of the compound in Ar (**a**) and N_2_ (**b**) matrices, compared with the spectra calculated for the *cis* and *trans* conformers at the B3LYP/aug-cc-pVTZ level of theory (**c**). Asterisks in frame (**a**) indicate expected spectral positions for the *cis* conformer (not detected in the Ar matrix).

Monomers of *m*FP were isolated in Ar (16 K) and N_2_ (14 K) matrices, and the mid-IR spectra (in the 3700–3500 cm^−1^ and 1550–600 cm^−1^ regions) were recorded immediately after the deposition of the matrices are presented in Figure 1a,b. Figure 1c shows the theoretical spectra of the two conformers, obtained from the B3LYP/aug-cc-pVTZ harmonic vibrational calculations.

The positions and intensities of the experimentally observed bands are listed in Table 2 and Table 3 for *trans* and *cis* conformers, respectively, and are compared with the computed vibrational data. Separation of the IR spectra of the two conformers was achieved thanks to the results of the broadband IR irradiations, which will be analyzed in Section 2.3. The last column of both tables includes a description of the vibrational modes based on the results of the PED analysis (see Table S2 for a definition of the internal coordinates).

**Figure 2:**
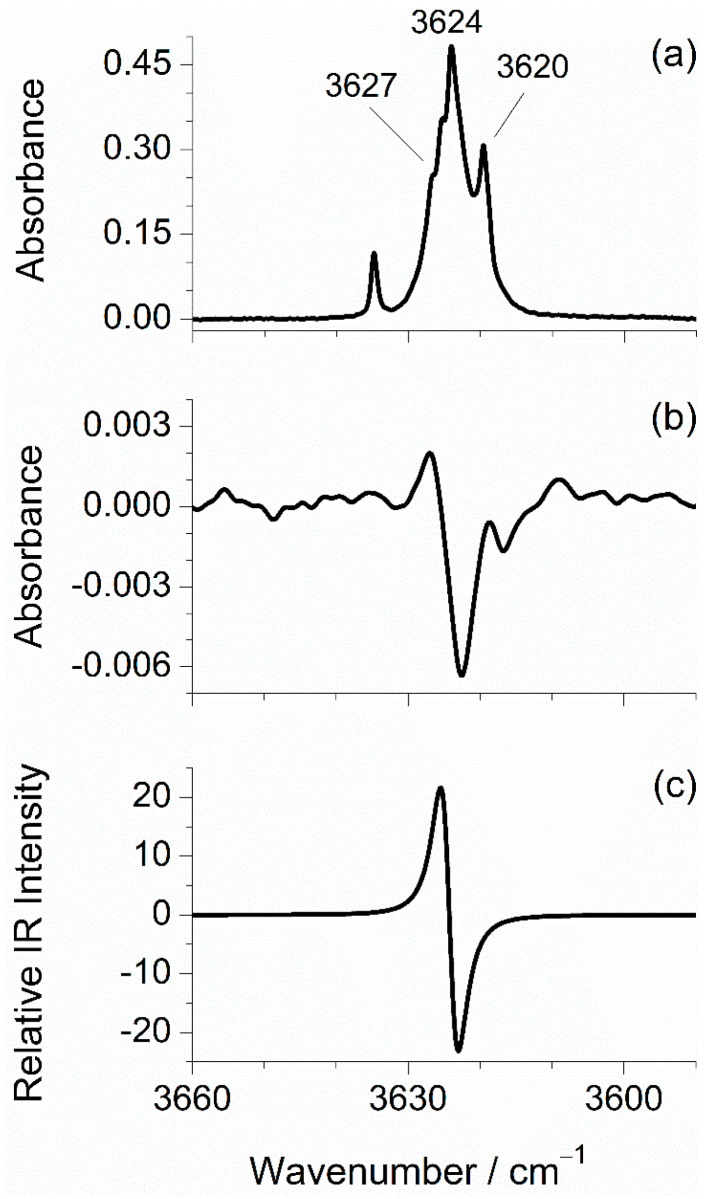
Spectral changes in the OH stretching region induced by broadband IR irradiations of *m*FP monomers in N_2_ matrix. (**a**) IR spectrum recorded immediately after removing the long-pass filter that cuts the IR radiation above ~2200 cm^−1^; (**b**) Difference spectrum obtained by subtracting the spectrum recorded immediately after removing the filter from that recorded after exposing the matrix-isolated compound to the whole range of broadband IR light emitted from the spectrometer source for 40 min (positive bands correspond to those growing up during exposition of the sample to the unfiltered IR light); (**c**) Simulated B3LYP/aug-cc-pVTZ difference spectrum: *cis* “minus” *trans*.

The strong feature located in the higher frequency region of the experimental mid-IR spectra is ascribed to the OH stretching vibration (νOH), see Figure 1 left, and Figure 2. This band has a multiplet profile, with the most intense component centered at 3638 cm^−1^ (Ar) or 3624 cm^−1^ (N_2_). The first wavenumber agrees with that previously reported in the literature under the same experimental conditions [12,14]. A slight red-shift of this band in the spectrum recorded in the N_2_ matrix, as compared to that recorded in Ar (Δν = ~14 cm^−1^, estimated from the positions of the most intense components), is consistent with the results of other matrix isolation studies performed for OH-containing compounds [37,40,41,42] and is explained by the establishment of hydrogen bond-like interactions between the OH group of *m*FP and the N_2_ matrix molecules. The spectral positions here reported for the νOH band are close to those observed in the IR spectra of matrix-isolated phenol (Ar: 3638–3631 cm^−1^ and N_2_: 3618 cm^−1^) [43,44] and of other substituted phenols with the OH group not involved in intramolecular hydrogen bonds (Ar: 3643–3637 cm^−1^ and N_2_: 3617–3615 cm^−1^) [19,22,23,24,25]. The OH stretching frequencies predicted for the two *m*FP conformers are very close to each other (3623.7 cm^−1^ for *trans* and 3624.8 cm^−1^ for *cis*). Thus, it is not possible, by simple spectral comparison, to assign any of the νOH band components to a specific conformer.

**Figure 3:**
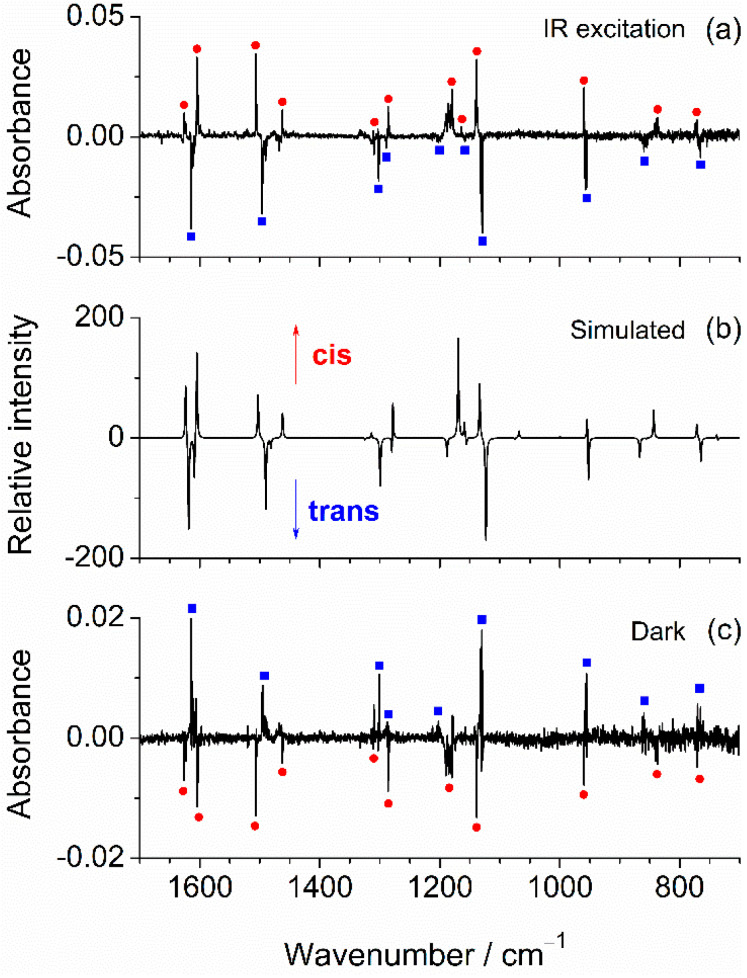
(**a**) Experimental difference IR spectrum of *m*FP monomers in N_2_ matrix, obtained by subtracting the spectrum recorded in the presence of a long-pass cut-off filter that is not transparent to the radiation above ~2200 cm^−1^, from that recorded after removing the filter and letting the sample exposed to the unfiltered radiation emitted from the spectrometer for ~40 min (positive bands are those growing up during exposition of the sample to the unfiltered IR light); (**b**) Simulated B3LYP/aug-cc-pVTZ difference spectrum: *cis* “minus” *trans*; (**c**) Experimental difference IR spectrum obtained by subtracting the spectrum recorded after the enrichment of the sample with the *cis* form from that recorded after keeping the sample in the dark for 90 min (positive bands correspond to those increasing during the time of permanence in the dark). Both spectra were collected in the presence of the long-pass filter.

In the fingerprint region of the IR spectra, some of the absorptions predicted for the two conformers are sufficiently separated from each other to allow identification of the individual conformers by comparison of the calculated and experimental spectra (see Figure 1 right, and Figure 3). This comparison shows that the features located in the spectra recorded in N_2_ at 1496, 1302, 1202, 1129, and 855 cm^−1^, and those recorded in Ar at 1495, 1298, 1202, 1126, and 853 cm^−1^ (bands marked with blue squares in the figure) are very well reproduced by the absorptions calculated for the most stable *trans* conformer at 1490.1 (νCC, δCH), 1299.3 (δCH), 1188.0 (δOH), 1123.1 (δCH), and 866.8 (γCH) cm^−1^, respectively. This good concordance reveals that this conformer was, as expected, trapped in both cryomatrices. On the other hand, the bands located in the spectrum recorded in N_2_ at 1506, 1463, 1179, 1140, and 839 cm^−1^ (marked with red circles) are very close to positions where only the *cis* conformer is expected to absorb: 1502.9 (νCC, δCH), 1462.0 (νCC, δCH), 1169.2 (δOH, δCH), 1133.5 (δCH), and 843.5 (γCH) cm^−1^, which strongly suggests that this conformer is also trapped in the as-deposited N_2_ matrix.

This set of bands, however, has no correspondence in the spectrum recorded in solid Ar (their expected positions in this spectrum are indicated by asterisks in Figure 1a), thus confirming our expectations that the *cis* conformer does not survive long enough in the Ar matrix to be spectroscopically detected by steady-state spectroscopy due to its fast depopulation (by H-atom tunneling) in favor of the most stable *trans* form.

Using the integrated absorbances (*A*) and respective calculated intensities (*I*) of the pair of bands observed at 1140 and 1129 cm^−1^ in the spectrum recorded in solid nitrogen, assigned, respectively, to the *cis* and *trans* conformers, the populations of the two rotamers in the as-deposited matrix were estimated by using the following formula:

[*cis*]/[*trans*] = (*A_cis_*/*A_trans_*) × (*I_trans_*/*I_cis_*).

The obtained values were [*trans*] = 54% and [*cis*] = 46%, which are close to the Boltzmann populations estimated for the gas phase prior to the matrix deposition. However, as will be seen below, this resemblance does not necessarily mean that the gas phase conformational equilibrium has been frozen in the low-temperature N_2_ matrix.

### 2.3. IR-Induced Conformational Changes

To definitely prove that the two *m*FP conformers are, in fact, present in the N_2_ matrix and in an attempt to separate their IR spectra, we have conducted a dedicated experiment where the matrix was deposited (by monitoring of the size of the growing matrix with a periodic recording of its IR spectrum) in the presence of a long-pass filter that protects the sample from the IR radiation above ~2200 cm^−1^. The idea of using the filter was to avoid the excitation of higher-frequency vibrations, in particular the OH stretching mode. Indeed, this mode can be expected to play a major role in a possible conformational photoswitching [45], because it is much more intense than all the other vibrations falling above 2200 cm^−1^, and also because the energy introduced in the molecule by excitation of this mode (~43 kJ mol^−1^) is more than twice of the energy barrier separating the two conformers. After the deposition had been completed, an IR spectrum was recorded in the presence of the filter. Subsequently, the filter was removed, and the matrix-isolated compound was left exposed to the unfiltered IR radiation of the Globar for ~40 min. The spectral changes resulting from removing the filter are evidenced in the difference spectrum shown in Figure 3a, in the 1700−700 cm^−1^ region. As one can see, the intensity of one set of bands increases (marked with red circles), while the intensity of another set of bands decreases (marked with blue squares). These spectral variations reveal the occurrence of changes in the relative populations of the two matrix-isolated conformers, thus irrefutably confirming that both rotamers are trapped in the N_2_ matrix. Comparison of the experimental difference spectrum with the one obtained by subtracting the spectrum calculated for *trans* from that calculated for *cis* (Figure 3b) shows very clearly that the decreasing bands are ascribable to the former *(trans*) conformer, while the growing ones are due to the latter (*cis*), i.e., the *cis*:*trans* ratio increases upon the exposition of the sample to the higher-frequency IR radiation of the spectrometer source. Spectral changes were also observed in the 3640–3605 cm^−1^ region and are depicted in Figure 2b. Although these changes are of smaller scale compared to those observed in the fingerprint region, they seem to indicate, by comparison with the calculated difference spectrum (Figure 2c), that the most intense component of the νOH multiplet with peaks at 3624/3620 cm^−1^ is due to the *trans* conformer, while the higher frequency shoulder at 3627 cm^−1^ originates in the *cis* conformer. Based on the above-described results, we were able to separate the individual spectral signatures of the two *m*FP conformers, which are given in Table 2 and Table 3.

**Figure 4:**
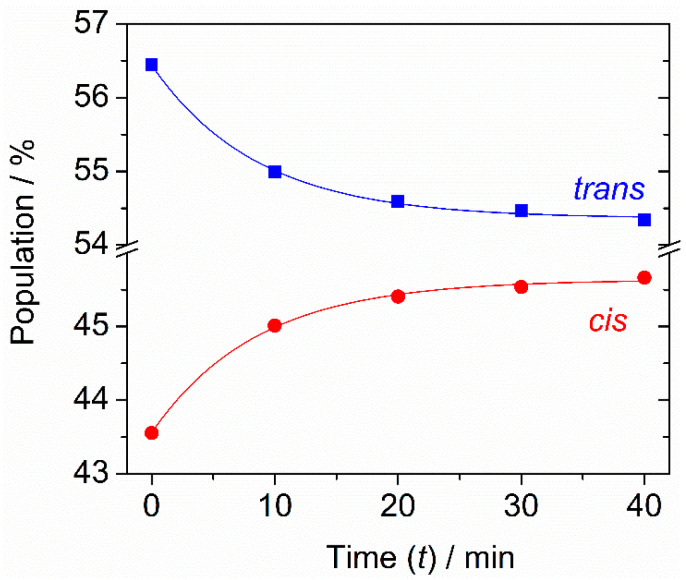
Time evolution of the population of the two conformers of *m*FP isolated in N_2_ at 14 K after removing the long-pass filter cutting the IR light above 2200 cm^−1^ emitted from the spectrometer and letting the sample be exposed to the unfiltered radiation. The populations of the conformers were estimated by integration of experimental IR intensity for the pair of bands observed at 1140 and 1129 cm^−1^, assigned, respectively, to the *cis* and *trans* conformers. Blue and red lines correspond to the fits of experimental data using the equation: *y* = *a* + *b* × *exp* [−(*k*_t→c_ + *k*_c→t_)*t*], describing the first-order reversible reaction. Fitting results: *a* = 54.36 ± 0.04 (blue squares) or *a* = 45.64 ± 0.04 (red circles); *b* = 2.08 ± 0.06 (blue squares) or *b* = −2.08 ± 0.06 (red circles); *k*_t→c_ + *k*_c→t_ = 0.12 ± 0.01; *R*^2^ = 0.997. On the other hand, *k*_c→t_/*k*_t→c_ = 54.36/45.64.

The evolution of the populations of the two conformers during the period of exposition of the matrix-isolated compound to the unfiltered IR radiation emitted from the spectrometer’s Globar was monitored, and the obtained results are shown in Figure 4. In the presence of the filter, the populations of the *trans* and *cis* conformers were estimated to be ~57% and ~43%, respectively, which match those estimated for the gas phase prior to deposition. This may have two explanations. It may indicate that excitation of the vibrational modes with wavenumbers lying between the lowest value of the calculated OH rotamerization barrier (~1195 cm^−1^) and the filter cut-off (~2200 cm^−1^) are not active in promoting conformational changes. Alternatively, and most likely, the estimated experimental populations must correspond to a photostationary state whose conformational distribution fortuitously coincides with that in the gas phase. When the filter was removed and the matrix-isolated conformers were exposed to the higher frequency range of the Globar radiation, the population of *cis* increased slightly while that of *trans* decreased concomitantly, until reaching a new photostationary state characterized by ~54% of *trans* and ~46% of *cis*. Not surprisingly, this conformational composition is the same as that estimated after the deposition of *m*FP in the N_2_ matrix without the presence of the filter. It should be noted that since the IR excitations are performed with broadband radiation, both conformers are vibrationally excited, meaning that the *trans* → *cis* and *cis* → *trans* photoisomerizations occur simultaneously. The experimental points obtained from the kinetic measurements were fitted to the equation of the first-order reversible reaction [22,23]:

*y* = *a* + *b × exp* [−(*k*_t→c_ + *k*_c→t_)*t*],

where *k*_t→c_ and *k*_c→t_ represent the rate constants corresponding to the *trans* → *cis* (*k*_t→c_) and *cis* → *trans* (*k*_c→t_) conversions. The following values were estimated from the fitting results: *k*_t→c_ = 8.9 × 10^−4^ s^−1^ and *k*_c→t_ = 1.1 × 10^−3^ s^−1^ (see the caption of Figure 4 for details). The small difference between the two rate constants is consistent with the fact that the populations of the two conformers in the photostationary state do not differ substantially, and this explains also why these populations are not very different from those existing in the gas phase.

### 2.4. Cis-to-Trans Tunneling Decay

The present paper is not concerned with the theory of tunneling. There are several recent reviews on the topic [46,47,48]. The approximate solutions for the equations describing the tunneling of a particle through a parabolic barrier were independently devised by Wentzel, Kramers, and Brillouin in 1926 [49,50,51], what has become known as the WKB approximate solution [52]. The probability *P* of tunneling through a parabolic barrier in the WKB model is described by the equation

$$P\left(E\right)=e^{-\pi^{2}w\sqrt{2mE}/h}$$

where a particle with mass *m* tunnels through a barrier with height *E* and width *w*, and *h* is the Planck constant.

**Figure 5:**
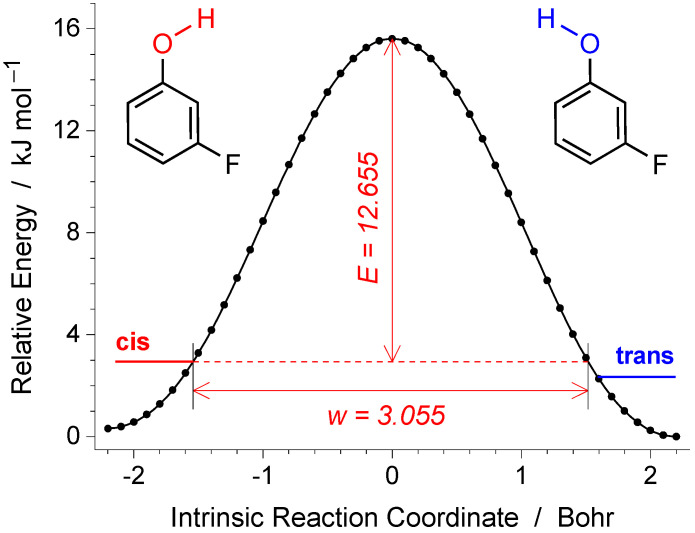
Intrinsic reaction coordinate (IRC) for the OH rotamerization in *m*FP computed at the B3LYP/6-31++G(d,p) level of theory in Cartesian (non-mass-weighted) coordinates. The vertical arrow (*E* = 12.655 kJ mol^−1^) designates the computed ZPE-corrected energy of the transition state with respect to the reactant (*cis*, given in red). The horizontal arrow designates the width (*w* = 3.055 Bohr) of the barrier at the ZPE level of the reactant. The relative zero energy corresponds to the product (*trans*, given in blue).

In order to characterize the possibility of tunneling OH rotamerization in *m*FP, we computed the reaction path for the flip of the OH group. Initially, the first-order transition state for the OH torsion was optimized, and the respective force constants were computed analytically. The intrinsic reaction path was then followed from the transition state in both directions and is presented in Figure 5. The intrinsic reaction coordinate (IRC) was set using the “IRC = Cartesian” option of the Gaussian software and is expressed in units of Bohr.

Using the calculated barrier height *E* (12.655 kJ mol^−1^) and width *w* (3.055 Bohr) for the *cis* → *trans* conversion, one can estimate the WKB transmission coefficient (tunneling probability) of the light H-atom (*m* = 1) in *m*FP as 1.83 × 10^−9^. The tunneling rate is a product of the transmission coefficient and the frequency of attempts. Assuming that the light H atom of the OH group is vibrating at an OH torsional frequency τ(OH)*_cis_*= 340.5 cm^−1^ (computed for the *cis* form), a tunneling rate constant of 1.87 × 10^4^ s^−1^ (a half-life time of 37 microseconds) was estimated for the *cis* → *trans* rotamerization. Such a fast decay, predicted for the tunneling of *cis m*FP, explains why this conformer has not been observed in the present experiments in the argon matrix. The observation of the *cis* form in the cryogenic nitrogen matrix, as mentioned above, should be related to the fact that the barrier for the OH flip in the N_2_ matrix increases considerably; however, a computational characterization of such a barrier is not trivial.

In order to experimentally verify the existence of H-tunneling for *m*FP isolated in N_2_, we carried out the following experiment. The N_2_ matrix was initially enriched with the *cis* form, by means of the exposition of the sample to the unfiltered light coming from the light source of the spectrometer. As shown in Section 2.3, this resulted in a sample with a *cis:trans* ratio of 46:54. Then, this sample was kept in complete darkness, by placing a metal plate between the optical window of the cryostat and the spectrometer. After 90 min in the dark, the metal plate was removed, and a new IR spectrum was immediately recorded in the presence of the long-pass filter. This resulted in decrease in the *cis:trans* ratio for the sample kept in the dark (40:60), as demonstrated in Figure 3c. Such a change in the conformational ratio can only be explained in terms of the tunneling mechanism. The action of recording the IR spectrum, however, very quickly cancels out the changes of conformational ratio resulting from tunneling, and registrations of subsequent spectra lead to a 43:57 *cis:trans* ratio, resulting from the effect of the IR spectrometer light source (below 2200 cm^−1^) on the sample, as described above.

As shown in Section 2.3, when *m*FP trapped in solid N_2_ is exposed to the unfiltered IR Globar light, the rate constant corresponding to the *cis → trans* conversion is estimated to be 1.1 × 10^−3^ s^−1^. It is important to note that this rate constant includes a contribution due to the over-the-barrier IR-induced rotamerization (which is expected to be dominant) and another one attributed to H-tunneling. Accordingly, the rate constant for the tunneling-induced *cis → trans* isomerization should be lower than 1.1 × 10^−3^ s^−1^. This is consistent with the results obtained for other substituted phenols studied in our laboratory (2-cyanophenol and 2-isocyanophenol) [22,23], for which the energy barriers calculated for the transformation of the higher into the lower energy rotamer (11–15 kJ mol^−1^) are close to the one computed in the present work. In fact, tunneling rate constants on the order of 10^−4^ s^−1^ were experimentally estimated for both compounds isolated in a low-temperature N_2_ matrix.

### 2.5. UV-Induced Transformations

The photochemistry of *m*FP was investigated by irradiating monomers of the compound isolated in an Ar matrix with UV laser light. The study of UV-induced changes in an argon matrix is simplified due to the absence of the higher-energy conformer of *m*FP. The irradiations were performed at *λ* = 280, 275, and 270 nm. This set of wavelengths falls within the absorption band observed in the ~260–280 nm region of the UV spectrum of *m*FP in cyclohexane solution [53]. After each irradiation, an infrared spectrum was collected to monitor the possible phototransformations taking place in the sample. Irradiations at *λ* = 280 and 275 nm did not result in any significant spectral changes. These were only observed upon exciting the matrix-isolated compound at the shortest wavelength (270 nm), which is close to the origin of the S_1_←S_0_ (π*←π) transition (271.5 nm) determined for the jet-cooled *trans* conformer of *m*FP by REMPI spectroscopy [54,55]. After 2 min of exposition of the Ar matrix to the UV laser light, a slight decrease in the bands ascribed to *m*FP was observed, corresponding to consumption of ~7% of the initial amount of this species. Concomitantly, a few new bands appeared in the IR spectrum, revealing the occurrence of photoreactions.

**Scheme 2:**
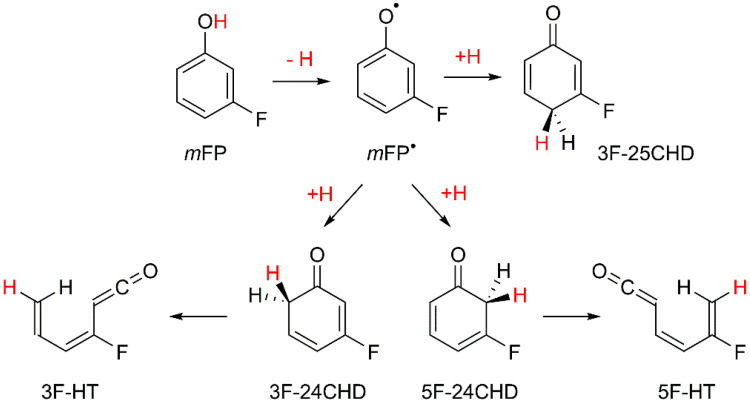
Isomerization pathways resulting from UV-irradiation of *m*FP isolated in an Ar matrix at 16 K (*λ* = 270 nm).

**Figure 6:**
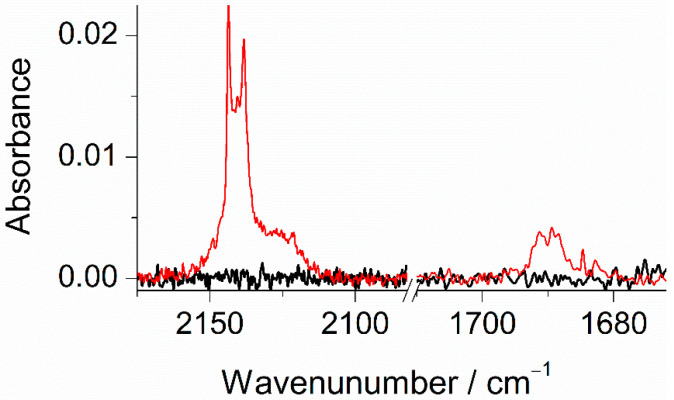
Selected regions of the experimental spectra of *m*FP (Ar, 16 K) recorded after 2 min of UV-irradiation at *λ* = 270 nm (red trace) and before any UV-irradiation (black trace).

Based on the results of previous photochemical studies carried out for matrix-isolated phenol [44], substituted phenols [24,25,56,57,58], and other structurally related compounds [59,60], the most important photoprocess following the UV excitations should be the breakage of the O–H bond in *m*FP, followed by reattachment of the released hydrogen atom at the carbon atoms at *ortho*- or *para*-positions with respect to the CO group, giving rise to fluoro-substituted cyclohexadienones, as seen in Scheme 2. The preference of the H-atom to recombine at these ring sites after its detachment from the OH group is theoretically predicted by natural spin density calculations for the *meta*-fluorophenoxyl radical (*m*FP^●^), which acts as an intermediate in the phototautomerization. The results of these calculations, carried out at the B3LYP/aug-cc-pVTZ level, are displayed in Figure S2. They are in line with those reported for analogous molecules [24,25,56] and clearly reveal that in addition to the oxygen atom, there are also other atoms with an appreciable amount of spin density. These are the two carbon atoms in *ortho*-positions and in the *para*-position, relative to the CO group and, therefore, these are the most likely positions of the *m*FP^●^ radical to recombine with the H-atom. Based on these theoretical arguments, the photoproducts resulting from the hydroxyl → oxo process must be 3-fluorocyclohexa-2,4-dien-1-one (3F-24CHD), 5-fluorocyclohexa-2,4-dien-1-one (5F-24CHD), and 3-fluorocyclohexa-2,5-dien-1-one (3F-25CHD), as shown in Figure S3. The presence of these species in the UV-irradiated Ar matrix is experimentally confirmed by the new band emerging at ~1690 cm^−1^ (see Figure 6), which is characteristic of the stretching vibration of a C=O group in cyclohexadienones [24,25,44,56,61]. This assignment is further corroborated by the results of the B3LYP/aug-cc-pVTZ vibrational calculations carried out for the three isomeric forms of fluorinated cyclohexadienones, where the wavenumbers predicted for the νC=O mode were found to fall between 1688 and 1701 cm^−1^ (see Figure S3 for details), thus nicely fitting the experimental value. In spite of the difficulties inherent to the experimental identification of the *m*FP^●^ radical, its presence in the UV-irradiated matrix is confirmed by the three very weak new bands at 1523, 1471, and 1172 cm^−1^, which appear close to the three unique strong absorptions predicted for this species at 1521.3, 1446.6, and 1166.2 cm^−1^, with IR intensities of 97.8, 50.2, and 106.9 km mol^−1^, respectively (see Figure S4).

A band with maxima at 2144/2138 cm^−1^ is also observed in the spectra recorded for the photolyzed Ar matrix (Figure 6). This new feature can be explained by the presence of compounds bearing a ketene (–C=C=O) fragment [44,62] and/or carbon monoxide [63,64]. Ketenes are generated from 3F-24CHD or 5F-24CHD by means of ring-opening reactions involving cleavage of the weaker C–C bond at the α-position with respect to the carbonyl group, i.e., that connecting this group to CH_2_, as seen in Scheme 2. This reaction is similar to the well-known ring-opening reaction of 1,3-cyclohexadiene yielding 1,3,5-hexatriene [65]. Note that in 3F-25CHD, the two C–C bonds at the α-position with respect to the C=O group are not particularly prone to be broken, meaning that this species is expected to be much less photoreactive than the other two isomers. Regarding carbon monoxide, and taking into consideration the results of our recent photochemical studies on alkyl-substituted phenols [24,25], its presence in the UV-irradiated matrix shall result from decarbonylation of 3F-24CHD or 5F-24CHD. The co-products of these photofragmentations are fluorinated cyclopentadienes. B3LYP/aug-cc-pVTZ vibrational calculations performed for these species (see Table S3) show the existence of strong absorptions in the fingerprint region, with IR intensities comparable to that predicted for monomeric carbon monoxide at the same level of theory (*I* = 79.9 km mol^−1^). This means that if CO contributes to the band observed at 2144/2138 cm^−1^, then the strong absorptions predicted for the co-produced fluorinated cyclopentadienes must also appear in the spectra recorded upon the UV irradiations. However, no such bands were observed, indicating that under the present experimental conditions, decarbonylation of fluorinated cyclohexadienones does not occur. Thus, the doublet band at 2144/2138 cm^−1^ is only attributed to open-ring ketenes 3-fluorohexa-1,3,5-trien-1-one (3F-HT) or 5-fluorohexa-1,3,5-trien-1-one (5F-HT), see Scheme 2. The wavenumbers (scaled) corresponding to the C=C=O antisymmetric stretching mode computed for the different configurational/conformational isomers adopted by these photoproducts were found to fall within the 2153–2134 cm^−1^ range, with calculated IR intensities in the range of 769–1556 km mol^−1^ (see Table S4). The remaining computed infrared intensities of the open-ring ketenes are at least an order of magnitude lower than those computed for the ν_as_(C=C=O) vibration, which prevented the identification of other bands due to these compounds in the spectra of the UV-irradiated Ar matrix.

## 3. Methods

### 3.1. Experimental Methods

Commercial *m*FP, obtained from TCI Europe N.V. (99%), was used in the matrix isolation experiments. Before usage, the liquid substance was additionally purified from volatile impurities by performing multiple freeze-pump-thaw cycles. Mixtures of *m*FP with the host matrix gas Ar (N60, Air Liquide) or N_2_ (N60, Air Liquide), were prepared in a 2 L glass reservoir using standard manometric procedures. The guest:host ratio was kept at ~1:600. The gaseous *m*FP/Ar and *m*FP/N_2_ mixtures were then allowed to slowly deposit onto a cold CsI window of a closed-cycle helium cryostat (APD Cryogenics, with a DE-202A expander) through a needle valve kept at ~298 K. The temperature of the cold window, 14 K (N_2_) or 16 K (Ar), was measured directly at the sample holder with an accuracy of 0.1 K by using a silicon diode sensor connected to a digital controller (Scientific Instruments, Model 9650-1). Deposition of the matrices was accompanied by a periodic recording of the IR spectra of the growing sample until it reached the desired size, which took approximately 3 h.

Mid-infrared spectra (4000–400 cm^−1^) of matrix-isolated *m*FP were recorded with a resolution of 0.5 cm^−1^ using a Thermo Nicolet 6700 Fourier-transform infrared (FTIR) spectrometer equipped with a Ge/KBr beam splitter and a deuterated triglycine sulfate (DTGS) detector. To protect the matrices from the higher frequency IR radiation, a standard Edmund Optics long-pass filter, with a transmission range below 2200 cm^−1^, was placed between the spectrometer light source (ETC EverGlo, Globar) and the sample. To completely suppress the broadband IR light of the spectrometer (dark conditions), a metal plate was placed between the light source and the cryostat. The single beam spectrum showing emission from the Globar, and the transmittance spectrum of the long-pass filter are provided in the Supplementary Materials (Figure S5).

IR-induced transformations were studied for the compound isolated in the N_2_ matrix. This was performed by depositing the compound in the presence of the long-pass filter followed by removal of the filter in order to allow the matrix-isolated compound to be exposed to the unfiltered IR radiation emitted from the Globar. UV-induced processes were also investigated by irradiating *m*FP isolated in solid Ar through the outer quartz window of the cryostat with monochromatic (spectral width = 0.2 cm^−1^) radiation from the 280–270 nm wavelength range. The frequency-doubled signal beam of a Quanta-Ray MOPO-SL optical parametric oscillator (OPO), pumped with a pulsed Nd:YAG laser (pulse duration 10 ns and repetition rate 10 Hz), was used for this purpose.

### 3.2. Computational Methods

All theoretical calculations were performed with the Gaussian 09 software package (Revision D.01) [66]. The geometries of the two *m*FP conformers and the photoproducts resulting from the UV irradiations were fully optimized at the DFT level of approximation by combining the diffuse-augmented Dunning’s correlation consistent triple-ζ basis set (aug-cc-pVTZ) [67,68] and the B3LYP hybrid functional, which employs the Becke three-parameter exchange functional [69], as well as the Lee, Yang, and Parr [70], and the Vosko, Wilk, and Nusair [71] correlation functionals. For *m*FP, additional full geometry optimizations were carried out by applying the Møller–Plesset (MP2) [72] method with the same basis set, and the quadratic configuration interaction with singles and doubles (QCISD) [73] method with the aug-cc-pVDZ basis set. Cartesian coordinates of the geometries of the two *m*FP conformers, optimized at the three levels of theory, are listed in Table S1. Subsequent to the full geometry optimizations, B3LYP and MP2 harmonic vibrational calculations were carried out for the geometries optimized at the respective levels. The nature of the obtained stationary points was checked through the analysis of the corresponding Hessian matrix. The wavenumbers and IR intensities extracted from the B3LYP harmonic vibrational calculations were used to help in the interpretation of the IR spectra. In order to account for the basis set limitations, the neglected part of electron correlation, and anharmonicity effects, the calculated wavenumbers were scaled down by factors of 0.950 and 0.980 for the regions above and below 3200 cm^−1^, respectively, as used in previous studies carried out in our laboratory [74].

The normal modes computed for *m*FP were analyzed in terms of a Potential Energy Distribution (PED). The force constants with respect to the Cartesian coordinates, computed at the B3LYP/aug-cc-pVTZ level of theory, were transformed to the force constants with respect to the molecule-fixed internal coordinates by solving the vibrational secular equation. This allowed ordinary normal coordinate analysis to be performed, following the procedure described by Schachtschneider and Mortimer [75]. The set of internal symmetry coordinates used in the PED analysis was defined as recommended by Pulay et al. [76] and is provided in Table S2.

## 4. Conclusions

In the present study, the structure, conformational space, and vibrational spectra for the monomers of matrix-isolated *meta*-fluorophenol, and photo-induced reactivity of the compound isolated in argon and nitrogen low-temperature matrices were investigated.

In the nitrogen matrix, both the most stable *trans* conformer and the higher energy *cis* form were observed experimentally by infrared spectroscopy, while in the argon matrix only the *trans* conformer was observed. In the argon matrix, the *cis* conformer undergoes fast spontaneous conversion by quantum mechanical tunneling to the *trans* form, following the general pattern found for other compounds. The occurrence of fast *cis → trans* tunneling in the argon matrix is also supported by WKB calculations on the isolated molecule of *m*FP, which predict a tunneling rate constant of 1.87 × 10^4^ s^−1^ (a half-life time of 37 microseconds) for the *cis* → *trans* rotamerization. Tunneling of the *cis* conformer into the *trans* form was also observed in the N_2_ matrix, but with a much lower probability; the upper limit for the rate constant for the tunneling-induced *cis → trans* isomerization in an N_2_ matrix being ca. 10^−3^ s^−1^. Since N_2_ molecules have a permanent quadrupole moment while noble Ar atoms do not, the existence of a nitrogen matrix solvation effect with the formation of −OH⋯N_2_ complexes is conceivable, leading to a stabilization of the *cis* conformer, which results in an increase in the *cis → trans* barrier height and width and, consequently, to a decrease in the tunneling probability [77].

We have also found that the relative populations of the two conformers present in an N_2_ matrix can be changed by infrared light. By collecting experimental spectra, either in the full mid-infrared range or only in the range below 2200 cm^−1^, we were able to observe changes in the relative band intensities, which demonstrated the effect of the higher frequency range (above 2200 cm^−1^) of the spectrometer’s light source on the relative population of the two conformers in the matrix. In the presence of a filter precluding the higher frequency IR light to reach the sample, the populations of the *trans* and *cis* conformers were estimated to be ~57% and ~43%, while in the absence of the filter they change to ~54% and ~46%, respectively. The kinetics of the infrared-induced processes (assuming simultaneous excitation of both conformers, leading to a different photostationary state after the removal of the filter, and ca. 40 min of exposition of the matrix to the full-range Globar infrared radiation) was followed and the rate constants corresponding to the *trans* → *cis* (*k*_t→c_) and *cis* → *trans* (*k*_c→t_) infrared-induced conversions were estimated as 8.9 × 10^−4^ s^−1^ and 1.1 × 10^−3^ s^−1^, respectively. The small difference between the two rate constants is consistent with the fact that the populations of the two conformers in the photostationary state do not differ substantially, and also with the fact that these populations are similar to those existing in the gas phase.

Both the experiments where infrared-induced conformational conversions were investigated, and those designed to observe the *cis* → *trans* tunneling decay in the N_2_ matrix, allowed us to reliably distinguish the bands originating from individual conformers in this matrix and perform the detailed assignment of the two sets of bands to the two forms.

Finally, the UV-induced phototransformations of *m*FP in an argon matrix were also studied. It was possible to conclude that the compound follows the general photochemistry of matrix-isolated phenols, starting with the photocleavage of the O–H bond, followed by recombination of the H atom at the *ortho-* and *para-* positions relative to the CO group of the initially formed fluoro-phenoxyl radical, giving rise to fluoro-substituted cyclohexadienones. The latter may then undergo a ring-opening reaction, involving cleavage of the weaker C−C bond at the α-position with respect to the carbonyl group, to generate ketene compounds. The vibrational signatures of the photogenerated fluoro-substituted cyclohexadienones and ketenes were successfully identified in the spectra of the irradiated matrices and, noteworthy, the same can be stated in relation to that of the phenoxyl radical produced in the first step of the observed photochemical reactions.

To sum up, in the case of the N_2_ matrix, the conformational populations of *m*FP observed in the experiment do not obey the true thermal equilibrium (at 15 K, the most stable *trans* conformer should dominate) and vary between [57% (*trans*), 43% (*cis*)] and [53% (*trans*), 47% (*cis*)]. Therefore, the additional importance of our work consists precisely in the demonstration that other factors contribute to the experimentally observed populations. These are (i) the quantum tunneling (the third reactivity paradigm [48]) and (ii) the vibrational excitation of the sample by the Globar source of the infrared spectrometer, the two factors which are frequently neglected during the analysis of experiments.

## Figures and Tables

**Table 1:** Relative energies (Δ*E*_el_, Δ*E*_0_, and Δ*G*), equilibrium populations (Pop.) at 298.15 K and dipole moments (*µ*/D) of the *cis* and *trans* conformers of *m*FP, and energy barriers for their interconversion (Δ*E*^‡^_el_), calculated at different levels of theory *^a^*.

	*trans*	*cis*
B3LYP/aug-cc-pVTZ		
Δ*E*_el_	0.00	0.58
Δ*E*_0_	0.00	0.69
Δ*G*	0.00	0.70
Pop.	57.0	43.0
*µ*	2.75	0.83
Δ*E*^‡^_el_	16.5	15.9
MP2/aug-cc-pVTZ (QCISD/aug-cc-pVDZ)		
Δ*E*_el_	0.00 (0.00)	0.70 (0.63)
Δ*E*_0_	0.00	0.75
Δ*G*	0.00	0.73
Pop.	57.3	42.7
*µ*	2.70 (3.11)	0.73 (0.78)
Δ*E*^‡^_el_	15.0	14.3

*^a^* All relative energies (kJ mol^−1^) are expressed with respect to the most stable *trans* conformer. Δ*E*_el_—relative electronic energy; Δ*E*_0_—relative electronic energy corrected for zero-point vibrational energy; and Δ*G*—relative Gibbs energy at 298 K. Equilibrium populations (Pop.) were estimated at 298.15 K by means of the Boltzmann distribution based on the Δ*G* values. The energy barriers (Δ*E*^‡^_el_, in kJ mol^−1^) are without the contribution of the zero-point vibrational energy and correspond to the energy difference between the first-order transition state and the respective local minimum.

**Table 2:** Bands observed in the IR spectrum of *m*FP isolated in nitrogen (N_2_) and argon (Ar) matrices, compared with the harmonic wavenumbers ($\tilde{v}$/cm^−1^), absolute intensities (*I*/km mol^−1^), and potential energy distribution (PED, %) calculated for the *trans* conformer of *m*FP at the B3LYP/aug-cc-pVTZ level of theory.

Experimental *^a^*		Calculated		Assignment, PED (%) *^c^*
N_2_, 14 K	Ar, 16 K	$\tilde{v}$ *^b^*	*I*	
		A′ modes		
**3624**/3620 (vs)	3639/**3638**/3632 (vs)	3623.7	69.2	νOH (100)
n.o.	n.o.	3147.8	<0.1	νC6H (100)
n.o.	n.o.	3146.9	1.0	νC4H (92)
3064 (vw)	3069/**3064** (vw)	3122.0	7.8	νC3H (81), νC2H (11)
3047 (vw)	3047 (vw)	3098.7	9.5	νC2H (89), νC3H (11)
1614 (vs)	1613 (vs)	1618.5	155.7	ν_c_CC (66)
**1612**/1611 (s)	1611 (s)	1609.2	71.3	ν_b_CC (70)
**1496**/1495 (s)	**1495**/1494 (vs)	1490.1	118.7	ν_d_CC (38), δC3H (26), δC6H (13), δC2H + δC4H (11)
**1492**/1489 (w)	**1489**/1488 (w)	1481.6	16.1	ν_e_CC (39), δC2H − δC4H (31), δOH (10)
1328/**1326** (w)	1325/**1324** (w)	1325.4	11.1	ν_f_CC (78), δC2H + δC4H (13)
1302 (s)	1299/**1298** (s)	1299.3	80.2	δC2H + δC4H (21), δC3H (16), δC6H (15), νCO (15), δOH (12)
1291/**1289** (m)	1286 (m)	1279.7	45.0	νCO (21), νC5F (17), ν_a_CC (15), δC2H − δC4H (12), δ_a_R (12)
1202 (m)	1202 (m)	1188.0	31.6	δOH (41), δC3H (16), νC5F (14)
1158 (w)	**1155**/1153 (w)	1155.4	13.5	δC2H + δC4H (38), δC3H (25), ν_f_CC (15), δOH (10)
1132/1130/**1129** (vs)	1128/1127/**1126** (vs)	1123.1	171.8	δC6H (43), νC5F (18), νCO (13)
n.o.	n.o.	1074.9	1.8	δC2H − δC4H (42), ν_e_CC (39)
**1005**/1004 (vw)	**1006**/1004 (vw)	998.9	4.5	δ_a_R (58), ν_a_CC (40)
958/**957**/955 (s)	958/**956**/955 (s)	952.3	78.4	ν_d_CC (40), νC5F (14), νCO (13)
740/**738** (vw)	**739**/738 (vw)	736.4	4.4	ν_a_CC (25), δ_c_R (20), δ_a_R (19), νC5F (12), νCO (11)
**532**/530 (vw)	534/**531** (vw)	528.1	3.8	δ_b_R (36), δ_c_R (28), νCO (11)
515 (vw)	516/**515** (vw)	511.3	5.8	δ_b_R (37), δ_c_R (35), νC5F (10)
477 (vw)	474/**473** (vw)	468.8	9.7	δCO + δCF (83)
n.i.	n.i.	324.5	1.4	δCO − δCF (80), ν_e_CC (12)
		A″ modes		
n.o.	n.o.	963.3	0.2	γ_c_CH (84)
862/860/**855**/854 (w)	862/859/857/**853** (m)	866.8	32.6	γ_b_CH (42), γ_a_CH (34), γCF (15)
n.o.	n.o.	855.9	1.7	γ_d_CH (70), γ_b_CH (27)
771/769/**766** (m)	766/**763**/760 (m)	764.7	38.9	γ_a_CH (39), γ_c_CH (17), γ_b_CH (15), γ_d_CH (13)
681/**680**/678 (w)	**679**/677 (w)	681.7	26.7	τ_a_R (87)
n.o.	n.o.	611.8	0.3	γCF (40), γCO (39), τ_b_R (14)
n.o.	n.o.	459.5	2.4	τ_c_R (59), τ_b_R (17), γCF (13), γCO (10)
n.i.	n.i.	343.9	94.7	τOH (99)
n.i.	n.i.	236.0	<0.1	τ_b_R (60), τ_c_R (33)
n.i.	n.i.	220.5	2.2	τ_a_R (23), γCO (22), τ_b_R (22), τ_c_R (18), γCF (16)

*^a^* Experimental wavenumbers are given in cm^−1^. Band intensities are expressed in qualitative terms: vs = very strong; s = strong; m = medium; w = weak; vw = very weak. For the multiplet bands, the most intense component is highlighted in bold. Assignment of the experimental bands to a specific conformer was supported by the spectral changes induced by the broadband IR irradiations and by experiments with the sample in the dark. Abbreviations: n.o. = not observed; n.i. = not investigated. *^b^* Calculated harmonic wavenumbers were multiplied by 0.950 (above 3200 cm^−1^) or 0.980 (below 3200 cm^−1^). *^c^* Definition of internal coordinates is given in the Supplementary Materials (see Table S2). PEDs lower than 10% are not included.

**Table 3:** Bands observed in the IR spectrum of *m*FP isolated in nitrogen (N_2_), compared with the harmonic wavenumbers ($\tilde{v}$/cm^−1^), absolute intensities (*I*/km mol^−1^), and potential energy distribution (PED, %) calculated for the *cis* conformer of *m*FP at the B3LYP/aug-cc-pVTZ level of theory.

Experimental *^a^*	Calculated		Assignment, PED (%) *^c^*
N_2_, 14 K	$\tilde{v}$ *^b^*	*I*	
	A′ modes		
3627 (vs)	3624.8	68.1	νOH (100)
n.o.	3147.0	0.8	νC4H (85), νC3H (10)
n.o.	3138.3	3.7	νC2H (83)
n.o.	3117.8	0.1	νC6H (71), νC3H (24)
3064 (vw)	3116.4	8.0	νC3H (60), νC6H (29)
1625 (s)	1623.6	91.5	ν_b_CC (56), ν_c_CC (16)
**1605**/1602 (vs)	1604.8	145.8	ν_c_CC (52), ν_b_CC (16)
1506 (s)	1502.9	73.2	ν_d_CC (36), δC3H (21), δC6H (14)
1463 (m)	1462.0	41.3	ν_e_CC (41), δC2H − δC4H (32)
1328/**1326** (vw)	1325.6	8.8	ν_f_CC (78), δOH (12)
1312 (vw)	1314.1	8.6	δC2H + δC4H (25), δC3H (17), δOH (14), νCO (13), δC6H (12)
**1286**/1285 (s)	1278.3	72.1	νCO (20), νC5F (19), ν_a_CC (15), δ_a_R (12), δC6H (11)
1190/1186/**1179** (vs)	1169.2	166.0	δOH (29), δC3H (26), ν_c_CC (11), νCO (10)
1164 (w)	1158.9	25.6	δOH (26), ν_f_CC (19), δC2H + δC4H (18), δC6H (15)
**1140**/1139/1137 (s)	1133.5	92.0	δC6H (29), δC2H + δC4H (28), νC5F (17)
1070 (vw)	1068.1	11.8	δC2H − δC4H (43), ν_e_CC (40)
**1005**/1004 (vw)	999.4	5.8	δ_a_R (57), ν_a_CC (41)
960 (m)	954.3	46.2	ν_d_CC (39), νC5F (15), νCO (14)
740/**738** (vw)	738.7	5.6	ν_a_CC (25), δ_c_R (20), δ_a_R (19), νC5F (12), νCO (11)
**532**/530 (vw)	527.0	4.1	δ_b_R (38), δ_c_R (26), νCO (11)
515 (vw)	511.0	7.5	δ_c_R (37), δ_b_R (37), νC5F (10)
477 (vw)	472.3	3.9	δCO + δCF (85)
n.i.	323.2	7.6	δCO − δCF (80), ν_e_CC (12)
	A″ modes		
n.o.	971.8	0.4	γ_c_CH (84)
n.o.	872.5	<0.1	γ_b_CH (60), γ_d_CH (40)
840/**839** (m)	843.5	46.8	γ_a_CH (44), γ_b_CH (15), γCO (15), γCF (14), γ_c_CH (12)
774 (w)	771.6	23.1	γ_a_CH (34), γ_d_CH (25), γ_c_CH (20), γ_b_CH (14)
681/**680**/678 (w)	681.8	23.7	τ_a_R (89)
n.o.	608.7	<0.1	γCF (40), γCO (37), τ_b_R (15)
456 (vw)	457.2	9.4	τ_c_R (59), τ_b_R (17), γCF (12), γCO (11)
n.i.	348.8	97.0	τOH (99)
n.i.	236.3	1.9	τ_b_R (58), τ_c_R (33), γCF (12)
n.i.	221.6	<0.1	τ_a_R (23), τ_b_R (23), γCO (22), τ_c_R (18), γCF (16)

*^a^* Experimental wavenumbers are given in cm^−1^. Band intensities are expressed in qualitative terms: vs = very strong; s = strong; m = medium; w = weak; vw = very weak. For the multiplet bands, the most intense component is highlighted in bold. Assignment of the experimental bands to a specific conformer was supported by the spectral changes induced by the broadband IR irradiations and by experiments with the sample in the dark. Abbreviations: n.o. = not observed; n.i. = not investigated. *^b^* Calculated harmonic wavenumbers were multiplied by 0.950 (above 3200 cm^−1^) or 0.980 (below 3200 cm^−1^). *^c^* Definition of internal coordinates is given in the Supplementary Materials (see Table S2). PEDs lower than 10% are not included.

## Data Availability

Not applicable.

## Supplementary Materials

The following supporting information can be downloaded at: https://www.mdpi.com/article/10.3390/molecules27238248/s1, Table S1, Cartesian coordinates for the *m*FP conformers fully optimized at different levels of theory; Table S2, Definition of the internal coordinates used in the normal mode analysis of *m*FP; Table S3, Results of harmonic vibrational calculations carried out for fluorinated cyclopentadienes; Table S4, Energetic and vibrational data computed for the different configurational/conformational isomers of the open-ring ketenes generated upon the UV photolysis of *m*FP; Figure S1, Relaxed potential energy scan calculated for *m*FP as function of the internal rotation of the O−H group; Figure S2, Spin density isosurface generated for the *meta*-fluorophenoxyl radical, including the values of the natural spin densities; Figure S3, Spectra simulated for the three isomeric forms of fluoro-substituted cyclohexadienones; Figure S4, Spectral indication of the photogeneration of the *meta*-fluorophenoxyl radical; Figure S5, Single beam spectrum showing emission of the Globar, and transmittance spectrum of the long-pass filter used in this work.
