# Supplementary material for: Infrared Spectra and Phototransformations of meta-Fluorophenol Isolated in Argon and Nitrogen Matrices
Source: Molecules. 2022 Nov 26;27(23):8248. doi: 10.3390/molecules27238248 (PMC9735537; doi:10.3390/molecules27238248)
Supplement: Supplementary file 1 [file molecules-27-08248-s001.zip › molecules-2016356-SI.pdf]

# Supplementary Material

## Infrared Spectra and Phototransformations of *meta*-Fluorophenol Isolated in Argon and Nitrogen Matrices

**A. J. Lopes Jesus [1,*], Juracy Regis de Lucena Júnior [2], Rui Fausto [3] and Igor Reva [3,4,*]**

[1] CQC-IMS, Faculty of Pharmacy, University of Coimbra, 3004-295 Coimbra,Portugal
[2] CCT, Department of Chemistry, State University of Paraíba, Campina Grande 58429-500, Brazil
[3] CQC-IMS, Department of Chemistry, University of Coimbra, 3004-535 Coimbra, Portugal
[4] CIEPQPF, Department of Chemical Engineering, University of Coimbra, 3030-790 Coimbra, Portugal
* Correspondence: ajorge@ff.uc.pt (A.J.L.J.); reva@eq.uc.pt (I.R.)

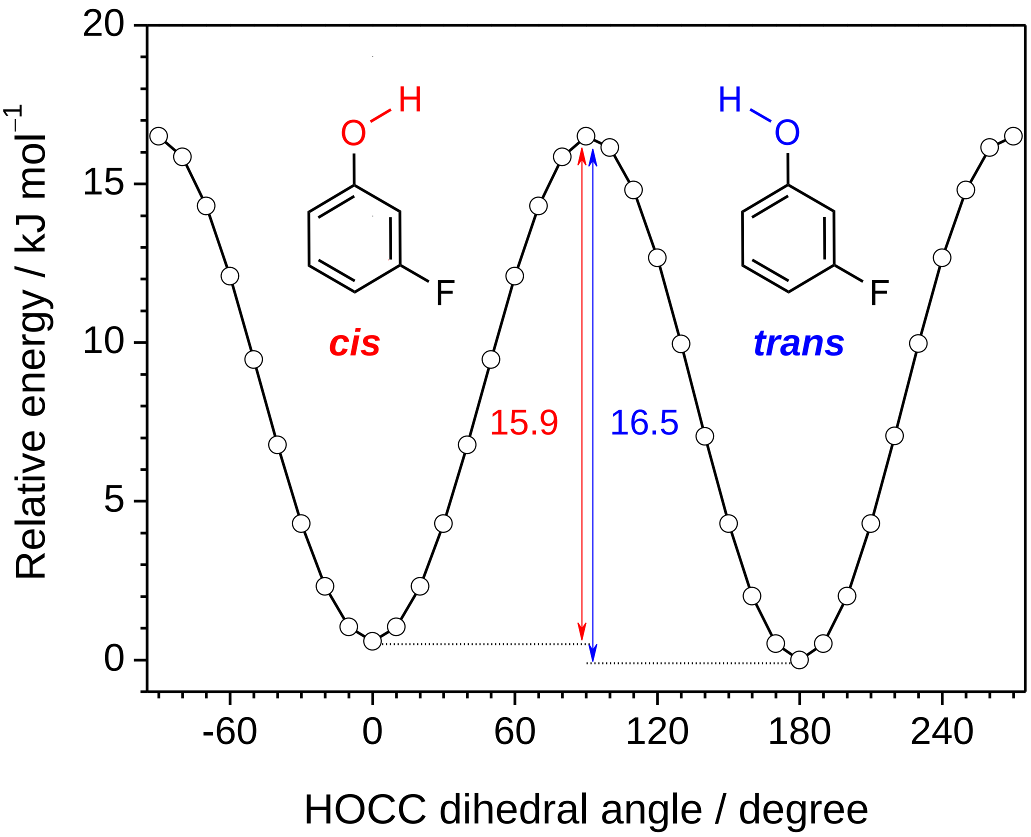


**Figure S1.** Relaxed potential energy scan calculated for *meta*-fluorophenol (*m*FP) as function of the internal rotation of the O−H group. The HOCC torsional angle was incrementally fixed in steps of 10° from −90° to +270° while all the remaining internal coordinates were optimized at the B3LYP/aug-cc-pVTZ level of theory. The values of *cis*-to-*trans* (red) and *trans*-to-*cis* (blue) isomerization barriers, calculated at the same level, are also included.

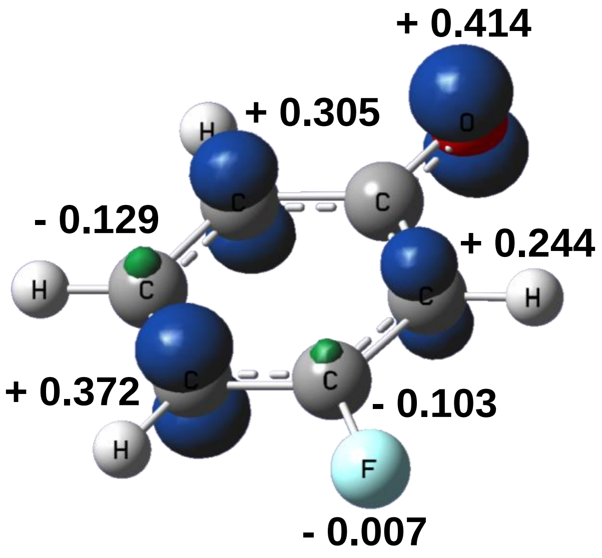


**Figure S2.** Spin density isosurface (isovalue = 0.015) generated for the *meta*-fluoro phenoxyl radical (blue indicates positive and green indicates negative spin densities), including the values of the natural spin densities calculated for the heavy atoms by means of a Natural Bond Orbital analysis carried out at the B3LYP/aug-cc-pVTZ level of theory.

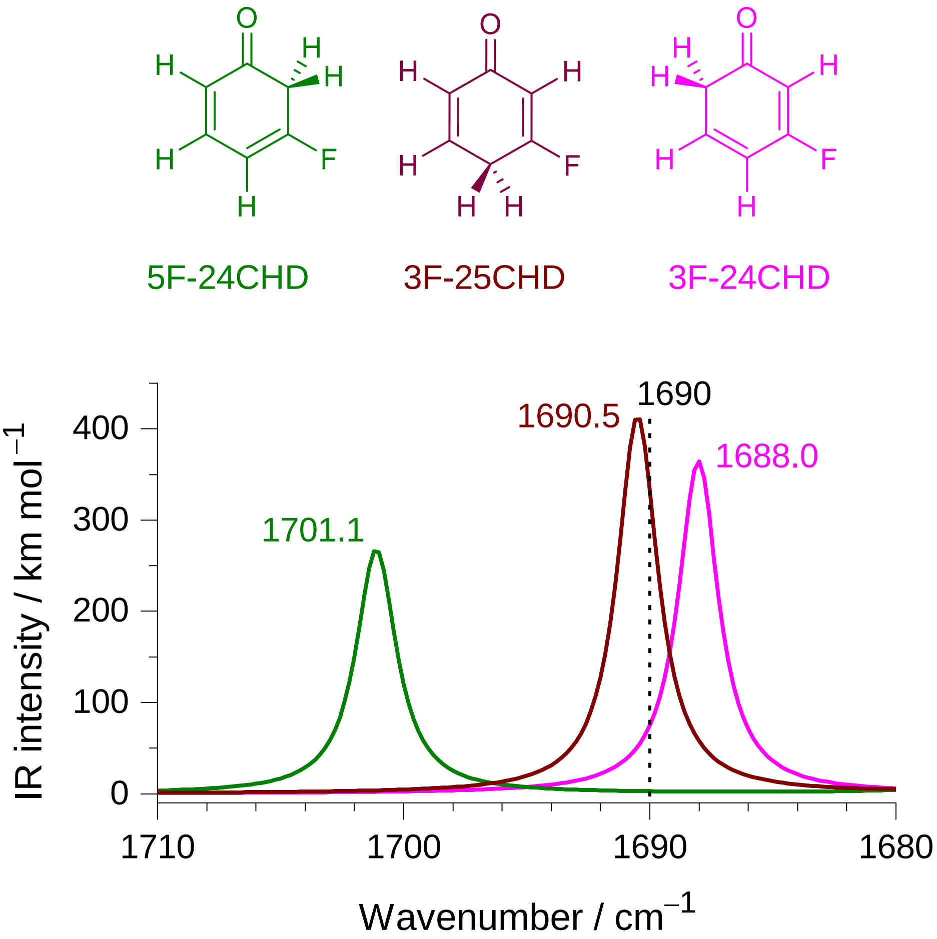


**Figure S3.** Spectra simulated for the experimentally relevant isomeric forms of fluoro-substituted cyclohexadienones (5F-24CHD, 5-fluorocyclohexa-2,4-dien-1-one; 3F-25CHD, 3-fluorocyclohexa-2,5-dien-1-one; 3F-24CHD, 3-fluorocyclohexa-2,4-dien-1-one; structures represented in the top row) in the 1710–1680 cm$^{-1}$ region, showing the bands corresponding to the C=O stretching vibration (νC=O). The vertical dotted line corresponds to the experimental position of the photoproduct band observed in the spectrum of *m*FP isolated in solid Ar, after irradiating the sample at λ = 270 nm (See Figure 6 of the main text).

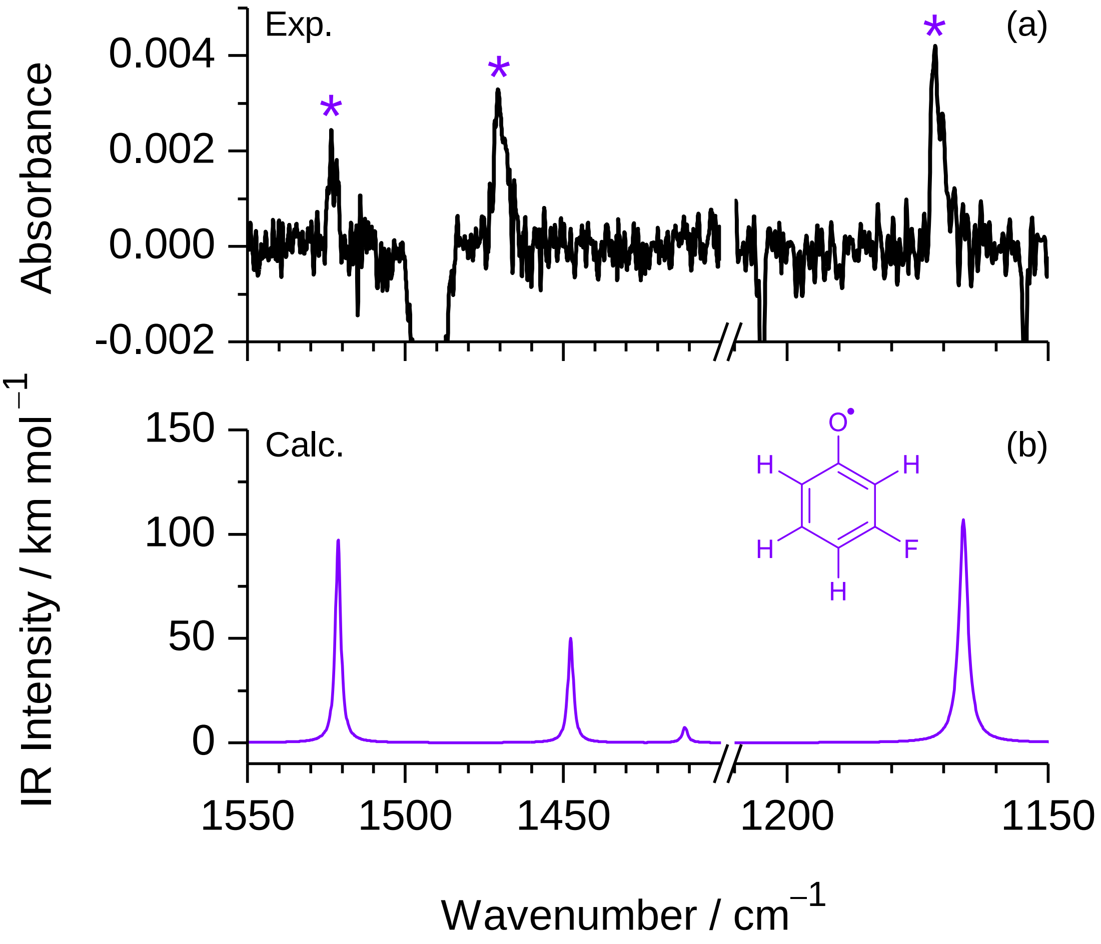


**Figure S4.** Spectral indication of the photogeneration of the *meta*-fluorophenoxyl radical. (a) Difference IR spectrum, obtained as the spectrum recorded after 2 min of UV-irradiation of the Ar/*m*FP matrix at $\lambda$ = 270 nm minus the spectrum recorded before the irradiation at this wavelength (positive bands are due to the photoproduct(s) that appear upon the irradiation while the negative ones are due to the consumed *m*FP reactant). (b) B3LYP/aug-cc-pVTZ simulated IR spectrum of the *m*FP$^{\bullet}$ radical.

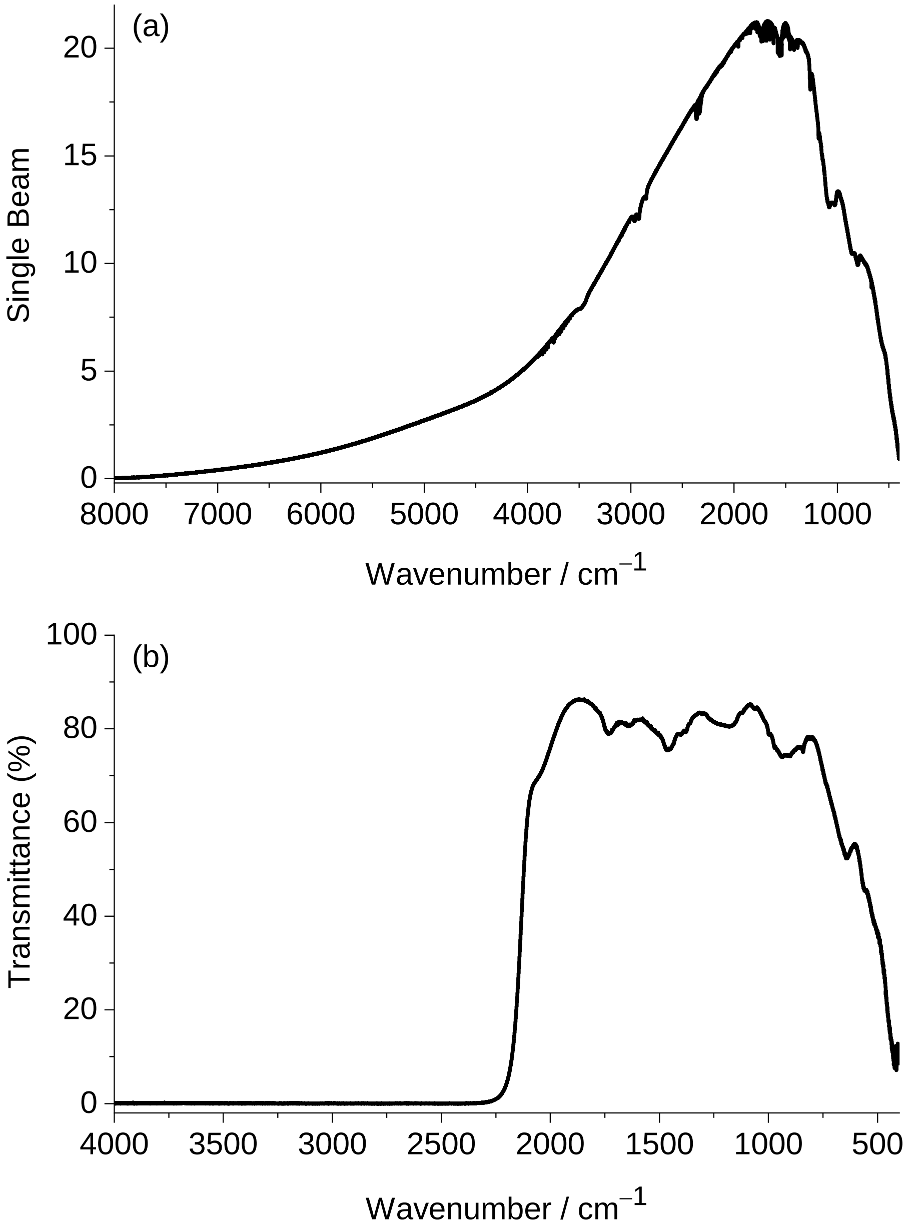


**Figure S5.** (a) Single beam spectrum recorded with the spectrometer Globar light source; (b) transmittance spectrum of the long-pass filter used in this work (transmission cut-off value at ~2200 cm$^{-1}$).

**Table S1**. Cartesian Coordinates (Å) for the *cis* and *trans* conformers of *meta*-fluorophenol (*m*FP) optimized at different levels of theory

| | | | | | | |
|---|---|---|---|---|---|---|
| | | | B3LYP/aug-cc-pVTZ | | | |
| | | *cis* | | | *trans* | |
| C | 1.196846 | 0.294599 | 0.000000 | 1.191506 | 0.299609 | 0.000000 |
| C | 1.187526 | -1.098795 | 0.000000 | 1.173614 | -1.093903 | 0.000000 |
| C | -0.024698 | -1.772669 | 0.000000 | -0.043501 | -1.764562 | 0.000000 |
| C | -1.235299 | -1.085007 | 0.000000 | -1.245852 | -1.068457 | 0.000000 |
| C | -1.187215 | 0.295678 | 0.000000 | -1.187457 | 0.315000 | 0.000000 |
| C | 0.000000 | 1.007138 | 0.000000 | 0.000000 | 1.020141 | 0.000000 |
| O | 2.412115 | 0.916431 | 0.000000 | 2.350487 | 1.020936 | 0.000000 |
| H | 2.295810 | 1.871641 | 0.000000 | 3.106079 | 0.424992 | 0.000000 |
| H | 2.127409 | -1.631065 | 0.000000 | 2.103653 | -1.648423 | 0.000000 |
| H | -0.029763 | -2.853934 | 0.000000 | -0.053914 | -2.845732 | 0.000000 |
| H | -2.186696 | -1.595047 | 0.000000 | -2.201174 | -1.571068 | 0.000000 |
| F | -2.345035 | 0.993608 | 0.000000 | -2.343800 | 1.014005 | 0.000000 |
| H | -0.021323 | 2.088823 | 0.000000 | 0.005799 | 2.099730 | 0.000000 |
| | | | MP2/aug-cc-pVTZ | | | |
| | | *cis* | | | *trans* | |
| C | 1.197319 | 0.295560 | 0.000000 | 1.192335 | 0.300436 | 0.000000 |
| C | 1.189173 | -1.098248 | 0.000000 | 1.176989 | -1.093810 | 0.000000 |
| C | -0.025250 | -1.775813 | 0.000000 | -0.041557 | -1.768858 | 0.000000 |
| C | -1.237438 | -1.086164 | 0.000000 | -1.246450 | -1.071333 | 0.000000 |
| C | -1.189691 | 0.296921 | 0.000000 | -1.189709 | 0.314016 | 0.000000 |
| C | 0.000000 | 1.008915 | 0.000000 | 0.000000 | 1.020754 | 0.000000 |
| O | 2.416237 | 0.914840 | 0.000000 | 2.349049 | 1.029336 | 0.000000 |
| H | 2.282880 | 1.869576 | 0.000000 | 3.097197 | 0.421259 | 0.000000 |
| H | 2.130905 | -1.628526 | 0.000000 | 2.108387 | -1.646886 | 0.000000 |
| H | -0.029398 | -2.857397 | 0.000000 | -0.050552 | -2.850401 | 0.000000 |
| H | -2.190062 | -1.594797 | 0.000000 | -2.202433 | -1.573612 | 0.000000 |
| F | -2.345099 | 0.994918 | 0.000000 | -2.344444 | 1.011853 | 0.000000 |
| H | -0.023005 | 2.091146 | 0.000000 | 0.005354 | 2.101055 | 0.000000 |
| | | | QCISD/aug-cc-pVDZ | | | |
| | | *cis* | | | *trans* | |
| C | 1.206307 | 0.299316 | 0.000000 | 1.202012 | 0.302894 | 0.000000 |
| C | 1.202245 | -1.108314 | 0.000000 | 1.190361 | -1.104014 | 0.000000 |
| C | -0.023584 | -1.791164 | 0.000000 | -0.041228 | -1.784207 | 0.000000 |
| C | -1.250160 | -1.097011 | 0.000000 | -1.259003 | -1.082592 | 0.000000 |
| C | -1.197475 | 0.297541 | 0.000000 | -1.196814 | 0.315675 | 0.000000 |
| C | 0.000000 | 1.022282 | 0.000000 | 0.000000 | 1.033817 | 0.000000 |
| O | 2.435405 | 0.922652 | 0.000000 | 2.367877 | 1.037674 | 0.000000 |
| H | 2.306675 | 1.879072 | 0.000000 | 3.119759 | 0.432674 | 0.000000 |
| H | 2.154558 | -1.643860 | 0.000000 | 2.131728 | -1.663416 | 0.000000 |
| H | -0.027610 | -2.885256 | 0.000000 | -0.049871 | -2.878232 | 0.000000 |
| H | -2.212331 | -1.613012 | 0.000000 | -2.224908 | -1.591598 | 0.000000 |
| F | -2.367298 | 1.003332 | 0.000000 | -2.366423 | 1.020467 | 0.000000 |
| H | -0.022844 | 2.115950 | 0.000000 | 0.006119 | 2.125523 | 0.000000 |

**Table S2.** Definition of the internal coordinates used in the normal mode analysis of *meta*-fluorophenol (*m*FP).

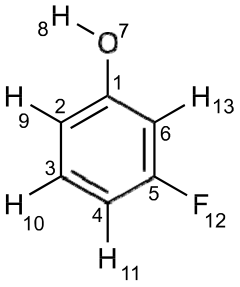


| Coordinate | Definition | Approximate description |
|---|---|---|
| $S_1$ | $(6^{-1/2})\ (r_{1,2} + r_{2,3} + r_{3,4} + r_{4,5} + r_{5,6} + r_{6,1})$ | $\nu_a$CC |
| $S_2$ | $(12^{-1/2})\ (2r_{1,2} - r_{2,3} - r_{3,4} + 2r_{4,5} - r_{5,6} - r_{6,1})$ | $\nu_b$CC |
| $S_3$ | $(4^{-1/2})\ (r_{2,3} - r_{3,4} + r_{5,6} - r_{6,1})$ | $\nu_c$CC |
| $S_4$ | $(12^{-1/2})\ (2r_{1,2} + r_{2,3} - r_{3,4} - 2r_{4,5} - r_{5,6} + r_{6,1})$ | $\nu_d$CC |
| $S_5$ | $(4^{-1/2})\ (r_{2,3} + r_{3,4} - r_{5,6} - r_{6,1})$ | $\nu_e$CC |
| $S_6$ | $(6^{-1/2})\ (r_{1,2} - r_{2,3} + r_{3,4} - r_{4,5} + r_{5,6} - r_{6,1})$ | $\nu_f$CC |
| $S_7$ | $r_{1,7}$ | $\nu$CO |
| $S_8$ | $r_{2,9}$ | $\nu$C2H |
| $S_9$ | $r_{3,10}$ | $\nu$C3H |
| $S_{10}$ | $r_{4,11}$ | $\nu$C4H |
| $S_{11}$ | $r_{5,12}$ | $\nu$C5F |
| $S_{12}$ | $r_{6,13}$ | $\nu$C6H |
| $S_{13}$ | $r_{7,8}$ | $\nu$OH |
| $S_{14}$ | $\beta_{8,1,7}$ | $\delta$OH |
| $S_{15}$ | $(4^{-1/2})\ (\beta_{7,6,1} - \beta_{7,2,1} + \beta_{12,4,5} - \beta_{12,6,5})$ | $\delta$CO + $\delta$CF |
| $S_{16}$ | $(4^{-1/2})\ (\beta_{7,6,1} - \beta_{7,2,1} - \beta_{12,4,5} + \beta_{12,6,5})$ | $\delta$CO − $\delta$CF |
| $S_{17}$ | $(2^{-1/2})\ (\beta_{10,2,3} - \beta_{10,4,3})$ | $\delta$C3H |
| $S_{18}$ | $(4^{-1/2})\ (\beta_{9,1,2} - \beta_{9,3,2} + \beta_{11,3,4} - \beta_{11,5,4})$ | $\delta$C2H + $\delta$C4H |
| $S_{19}$ | $(4^{-1/2})\ (\beta_{9,1,2} - \beta_{9,3,2} - \beta_{11,3,4} + \beta_{11,5,4})$ | $\delta$C2H − $\delta$C4H |
| $S_{20}$ | $(2^{-1/2})\ (\beta_{13,5,6} - \beta_{13,1,6})$ | $\delta$C6H |
| $S_{21}$ | $(6^{-1/2})\ (\beta_{6,2,1} - \beta_{1,3,2} + \beta_{2,4,3} - \beta_{3,5,4} + \beta_{4,6,5} - \beta_{5,1,6})$ | $\delta_a$R |
| $S_{22}$ | $(12^{-1/2})\ (2\beta_{6,2,1} - \beta_{1,3,2} - \beta_{2,4,3} + 2\beta_{3,5,4} - \beta_{4,6,5} - \beta_{5,1,6})$ | $\delta_b$R |
| $S_{23}$ | $(4^{-1/2})\ (\beta_{1,3,2} - \beta_{2,4,3} + \beta_{4,6,5} - \beta_{5,1,6})$ | $\delta_c$R |
| $S_{24}$ | $(6^{-1/2})\ (\tau_{4,3,2,1} - \tau_{5,4,3,2} + \tau_{6,5,4,3} - \tau_{1,6,5,4} + \tau_{2,1,6,5} - \tau_{3.2,1,6})$ | $\tau_a$R |
| $S_{25}$ | $(4^{-1/2})\ (\tau_{5,4,3,2} - \tau_{6,5,4,3} + \tau_{2,1,6,5} - \tau_{3.2,1,6})$ | $\tau_b$R |
| $S_{26}$ | $(12^{-1/2})\ (-\tau_{3.2,1,6} + 2\tau_{4,3,2,1} - \tau_{5,4,3,2} - \tau_{6,5,4,3} + 2\tau_{1,6,5,4} - \tau_{2,1,6,5})$ | $\tau_c$R |
| $S_{27}$ | $(2^{-1/2})\ (\tau_{8,7,1,6} + \tau_{8,7,1,2})$ | $\tau$OH |
| $S_{28}$ | $\gamma_{7,6,1,2}$ | $\gamma$CO |
| $S_{29}$ | $(4^{-1/2})\ (\gamma_{9,1,2,3} + \gamma_{10,2,3,4} + \gamma_{11,3,4,5} + \gamma_{13.5,6,1})$ | $\gamma_a$CH |
| $S_{30}$ | $(4^{-1/2})\ (\gamma_{9,1,2,3} + \gamma_{10,2,3,4} - \gamma_{11,3,4,5} - \gamma_{13.5,6,1})$ | $\gamma_b$CH |
| $S_{31}$ | $(4^{-1/2})\ (\gamma_{9,1,2,3} - \gamma_{10,2,3,4} + \gamma_{11,3,4,5} - \gamma_{13,5,6,1})$ | $\gamma_c$CH |
| $S_{32}$ | $\gamma_{12,4,5,6}$ | $\gamma$CF |
| $S_{33}$ | $(4^{-1/2})\ (\gamma_{9,1,2,3} - \gamma_{10,2,3,4} - \gamma_{11,3,4,5} + \gamma_{13,5,6,1})$ | $\gamma_d$CH |

Abbreviations: $\nu$ − stretching, $\delta$ – in-plane bending, $\gamma$ − out-of-plane bending, $\tau$ – torsion, R – ring. See figure above for the atom numbering; $r_{i,j}$ is the distance between atoms $A_i$ and $A_j$; $\beta_{i,j,k}$ is the angle between vectors $A_kA_i$ and $A_kA_j$; $\tau_{i,j,k,l}$ is the dihedral angle between the plane defined by $A_i$, $A_j$, $A_k$ and the plane defined by $A_j$, $A_k$, $A_l$ atoms; $\gamma_{i,j,k,l}$ is the angle between the vector $A_kA_i$ and the plane defined by atoms $A_j$, $A_k$, $A_l$. The combinations [(+), (+)] and [(+), (−)] denote in-phase and in-opposite-phase couplings between coordinates of different types.

**Table S3.** Wavenumbers ($\tilde{\nu}$ / cm$^{-1}$) and IR intensities ($I$/ km mol$^{-1}$) extracted from B3LYP/aug-cc-pVTZ harmonic vibrational calculations carried out for fluorinated cyclopentadienes on geometries optimized at the same level. [a]

| 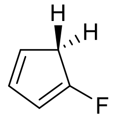<br>1-fluorocyclopenta-1,3-diene | | 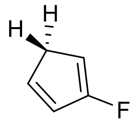<br>2-fluorocyclopenta-1,3-diene | |
|---|---|---|---|
| $\tilde{\nu}$ | $I$ | $\tilde{\nu}$ | $I$ |
| 3168.5 | 0.7 | 3177.6 | 0.8 |
| 3162.5 | 8.1 | 3165.1 | 3.8 |
| 3135.7 | 7.4 | 3144.7 | 2.0 |
| 2992.7 | 2.8 | 2974.6 | 5.3 |
| 2966.8 | 4.9 | 2954.8 | 9.8 |
| **1640.0** | **88.7** | **1641.1** | **94.0** |
| **1546.5** | **53.1** | 1543.2 | 10.9 |
| 1392.8 | 18.3 | 1390.9 | 4.7 |
| 1358.7 | 39.6 | **1376.1** | **67.2** |
| **1299.6** | **59.3** | 1261.9 | 34.2 |
| 1232.9 | 7.7 | 1228.8 | 11.3 |
| 1179.1 | 25.5 | **1205.5** | **52.3** |
| 1104.2 | 0.6 | 1109.7 | 0.3 |
| 1102.6 | 0.8 | 1068.7 | 6.7 |
| 980.0 | 2.6 | 975.5 | 10.0 |
| 940.3 | 0.8 | 937.5 | 0.1 |
| 936.5 | 12.5 | 923.2 | 28.8 |
| 901.8 | 14.1 | 903.8 | 6.1 |
| 894.8 | 15.4 | 902.6 | 26.5 |
| 850.7 | 21.7 | 793.8 | 2.4 |
| 794.1 | 5.9 | **768.2** | **41.5** |
| **670.3** | **50.1** | 675.4 | 3.1 |
| 606.8 | 24.8 | 608.9 | 12.4 |
| 494.7 | 1.7 | 594.4 | 22.6 |
| 392.1 | 3.3 | 400.5 | 1.3 |
| 389.5 | 8.2 | 373.8 | 3.1 |
| 255.0 | 1.3 | 258.7 | 1.6 |

[a] Wavenumbers were scaled by 0.980. The most intense modes, with computed IR intensities over 40 km mol$^{-1}$, are highlighted in bold.

**Table S4.** Energetic and vibrational data calculated at the B3LYP/aug-cc-pVTZ level for the different structures of the open-ring ketenes 3-fluorohexa-1,3,5-trien-1-one (3F-HT) and 5-fluorohexa-1,3,5-trien-1-one (5F-HT).[a]

| Structure | $\Delta E_0$ / kJ mol$^{-1}$ | $\nu_{as}$(C=C=O) / cm$^{-1}$ | $I$ / km mol$^{-1}$ |
|---|---|---|---|
| **3F-HT** | | | |
| 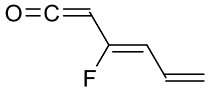 | 8.77 | 2150.3 | 1448.6 |
| 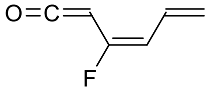 | 11.50 | 2152.7 | 1397.8 |
| 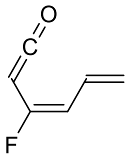 | 19.96 | 2147.7 | 768.7 |
| 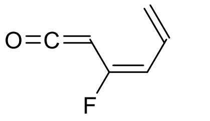 | 25.82 | 2151.5 | 1271.0 |
| **5F-HT** | | | |
| 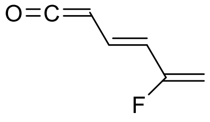 | 0.00 [b] | 2136.7 | 1535.8 |
| 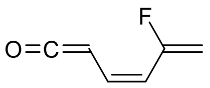 | 6.17 | 2134.1 | 1555.7 |
| 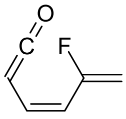 | 16.03 | 2134.6 | 984.3 |
| 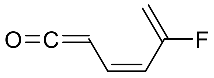 | 17.06 | 2138.0 | 1339.3 |

[a] $\Delta E_0$ = Relative zero-point corrected energies. Wavenumbers were scaled by 0.974 (obtained for this spectral range in our recent work, see Ref. [56]). [b] Most stable open-ring ketene isomer among those presented here. The absolute zero-point corrected energy of this isomer, computed at the B3LYP/aug-cc-pVTZ level is equal to −406.716449 hartree.
